# The neglected perioperative population of undiagnosed diabetics – a retrospective cohort study

**DOI:** 10.1186/s12893-020-00844-2

**Published:** 2020-08-18

**Authors:** Wei W. Teo, Lian K. Ti, Lyn L. Lean, Edwin Seet, Ambika Paramasivan, Weiling Liu, Jiexun Wang, Vanessa Chua, Lydia Q. Liew

**Affiliations:** 1 Department of Anaesthesia, National University Health System, 5 Lower Kent Ridge Road, Singapore, 119074 Singapore; 2 Department of Anaesthesia, Khoo Teck Puat Hospital, 90 Yishun Central, Singapore, 768828 Singapore

**Keywords:** Diabetes mellitus, General surgery, Morbidity, Mortality, Asian

## Abstract

**Background:**

Diabetes is known to increase morbidity and 30-day mortality in adults undergoing non-cardiac surgery, but longer term outcomes are less studied. This study was done to explore how undiagnosed and known diabetes affect 30-day and one-year morbidity and mortality outcomes. The secondary aim was to study the prevalence of undiagnosed diabetics in our perioperative Asian surgical population.

**Methods:**

A retrospective cohort study of 2106 patients aged > 45 years undergoing non-cardiac surgery in a single tertiary hospital was performed. Undiagnosed diabetics were identified (HbA1c ≥6.5% or fasting blood glucose ≥126 mg/dL) and relevant demographic, clinical and surgical data were analyzed to elicit the relationship to adverse outcomes. Univariate analysis was first performed to identify significant variables with *p*-values ≤0.1, which were then analyzed using multiple logistic regression to calculate the adjusted odds ratio.

**Results:**

The prevalence of undiagnosed diabetes was 7.4%. The mean and median HbA1c of known diabetics were 7.9 and 7.5%, while the mean and median HbA1c for undiagnosed diabetics were 7.2 and 6.8% respectively. 36.4% of known diabetics and 20.5% of undiagnosed diabetics respectively had a random blood glucose > 200 mg/dL. Undiagnosed diabetics had a three-fold increase in 1-year mortality compared to non-diabetics (adjusted OR 3.46(1.80–6.49) *p* < 0.001) but this relationship was not significant between known and non-diabetics. Compared to non-diabetics, known diabetics were at increased risks of new-onset atrial fibrillation (aOR 2.48(1.01–6.25) *p* = 0.047), infection (aOR 1.49(1.07–2.07) *p* = 0.017), 30-day readmission (aOR 1.62(1.17–2.25) *p* = 0.004) and 30-day mortality (aOR 3.11(1.16–8.56) *p* = 0.025).

**Conclusions:**

Although undiagnosed diabetics have biochemically less severe disease compared to known diabetics at the point of testing, they are at a one-year mortality disadvantage which is not seen among known diabetics. This worrying trend highlights the importance of identifying and treating diabetes. Congruent to previous studies, known diabetics have higher morbidity and 30-day mortality compared to non-diabetics.

## Background

Type 2 diabetes mellitus is one of the most prevalent and morbid conditions affecting the lives of over 400 million people worldwide [1]. Its prevalence is estimated to double within 20 years in the United States alone [2]. The disease is a major cause of microvascular and macrovascular complications including blindness, renal failure, cardiovascular and cerebrovascular events, with significant impact on quality of life and life expectancy.

More than 200 million adults undergo major non-cardiac surgery every year, and a significant proportion have diabetes [3]. Numerous studies have demonstrated a clear association between perioperative hyperglycemia and diabetes with increased morbidity related outcomes including higher rates of infection and longer hospital length of stay [4–8]. An observational study also demonstrated significant association between increased perioperative glucose level and mortality risk amongst patients without prior diagnosis of diabetes, but not the known diabetics [9]. However the results are to be interpreted with caution as a significant proportion of the hyperglycaemic patients included could have actually been underdiagnosed diabetics. A previous large retrospective study on a Western population had shown the prevalence of undiagnosed diabetes to be a significant 10% of the surgical population [10]. However to date, there is a paucity of studies looking into the implications on perioperative morbidity and mortality, regardless of the patient’s preoperative glycemic status. Although many studies agree that diabetes increases postoperative morbidity and 30-day mortality, it has not been shown to affect 1-year mortality outcomes [4, 5, 11–13].

An epidemiological study in 2013 estimated that there are 170 million cases of undiagnosed diabetes globally while another prospective study showed a prevalence of 13.4% of undiagnosed diabetes in their intensive care unit [14]. Diagnosis of diabetes remains challenging due to the often insidious onset of symptoms, low disease awareness amongst the public and limited healthcare resources for screening. Asians have a different metabolic profile and adipose distribution that has seen a shift in the definition of obesity between Asians and Westerners. This shift could potentially be applicable to diabetes and account for differences in demographics, risk factors and disease trajectory, and could explain the increased prevalence of diabetes in Asians [15]. Furthermore, most screening criteria for diabetes are largely developed by Westerners that may not be specific to the Asian population.

This study was done primarily to explore how undiagnosed and known diabetes affect both 30-day and 1-year morbidity and mortality outcomes when compared to non-diabetics. The secondary outcome was to look at the prevalence of undiagnosed diabetics in our perioperative Asian surgical population.

## Methods

Following ethics committee approval from the Institutional Review Board (National Healthcare Group Domain Specific Review Board reference: 2016/01273), a retrospective cohort study of 2300 patients aged 45 years old and above undergoing non-cardiac surgery from January 2015 to July 2015 was performed in a university-affiliated tertiary hospital.

Patients undergoing surgery were identified through operating theatre audit records which capture > 90% of patients undergoing any form of surgery. Members of the research team used International Classification of Diseases coding and manually reviewed each patient’s electronic medical records to obtain relevant information pertaining to their baseline characteristics, co-morbidities and nature of surgery.

The inclusion criteria included patients 45 years old and above undergoing intermediate or high-risk non-cardiac surgery, defined as surgery requiring at least 23 h stay in hospital. Patients undergoing multiple surgeries had only the index surgery considered, with their subsequent surgeries excluded from the analysis, resulting in a total of 2106 patient encounters analysed.

Relevant demographic, clinical and surgical data were analysed to elicit their relationship to mortality at 1 year after surgery. The presence or absence of patient comorbidities were considered positive if they were known to be present at the time of surgery as indicated by anaesthetic records. These records are routinely obtained by direct patient questioning as well as by searching electronic hospital records. Laboratory investigations were analysed if they were performed within 6 months prior to surgery with no change in patient’s medical status in between. If there were more than one of the same investigation performed in the same patient, the one done closest to the time of surgery was used for analysis. All perioperative factors studied are shown in Table 1.

**Table 1:** Table showing the demographic, clinical and surgical data of the population studied

Demographics		Known DM (***n*** = 602)	Undiagnosed DM (***n*** = 155)	No DM (***n*** = 1349)	***p***-value
**Male (%)**		324 (53.8)	91 (58.7)	712 (52.8)	0.37
**Age, mean**		64.9 (SD^b^ 10.3)	64.7 (SD 11.3)	63.0 (SD 10.9)	< 0.001
**Ethnicity**	**Chinese (*n*** **= 1479) (%)**	356 (59.1)	95 (61.3)	1028 (76.2)	< 0.001
	**Malay (*n*** **= 402) (%)**	148 (24.6)	41 (26.5)	213 (15.8)	
	**Indian (*n*** **= 178) (%)**	81 (13.5)	17 (11.0)	80 (5.9)	
	**Others (*n*** **= 47) (%)**	17 (2.8)	2 (1.3)	28 (2.1)	
**BMI, mean**		26.5 (SD 5.67)	25.6 (SD 6.2)	24.9 (SD 5.1)	< 0.001
**Type of Anaesthesia**	**GA (%)**	420 (77.9)	124 (86.1)	1153 (90.0)	< 0.001
	**RA (%)**	71 (13.2)	15 (10.4)	87 (6.8)	
	**MAC (%)**	48 (8.9)	5 (3.5)	41 (3.2)	
**Emergency case (%)**		213 (35.4)	63 (40.6)	315 (23.4)	< 0.001
**ASA**^**a**^ **Score**	**ASA 1 (%)**	NA	16 (10.3)	174 (12.9)	< 0.001
	**ASA 2 (%)**	253 (42.0)	45 (29.0)	655 (48.6)	
	**ASA 3 (%)**	321 (53.3)	71 (45.8)	445 (33.0)	
	**ASA 4 (%)**	44 (7.3)	23 (14.8)	74 (5.5)	
**John Hopkins Surgical** **Severity Criteria**	**1 (%)**	258 (43.3)	45 (30.0)	382 (28.6)	< 0.001
	**2 (%)**	262 (44.0)	51 (34.0)	728 (54.5)	
	**3 (%)**	76 (12.8)	54 (36.0)	225 (16.9)	

^a^*ASA* American Society of Anesthesiologists classification

^b^*SD* Standard deviation

By definition of ASA, diabetics are considered to be > ASA 2

The adverse outcomes investigated were mortality at 30 days, mortality at 1 year, new onset atrial fibrillation, development of any infection including urinary tract, lung, line and surgical site, as well as readmission within 30 days. The outcomes were defined to be positive if it developed within 30 days of the surgical date as indicated by the primary team using ICD-9 coding or if indicated in the discharge summary documentation. Mortality was determined using electronic medical records that is linked to our national health registry. This registry includes information from all primary healthcare services and hospitals in Singapore. If the patient has deceased, this would be indicated on the record, including the date of death. Proof that a patient was alive was confirmed by searching for evidence of any subsequent healthcare visits, prescriptions, laboratory and radiological investigations within the time period.

Patients were stratified into the following categories: known diabetes (as indicated on the anesthesia records), undiagnosed diabetes (based on a fasting blood glucose ≥126 mg/dL or HbA1c ≥6.5% in patients without a prior diagnosis of diabetes), or no diabetes (as indicated on the anesthesia records or those who had no records of fasting blood glucose or HbA1c) [14, 16]. Fasting blood glucose values were from samples drawn from patients kept nil per os for a minimum of 8 h. These samples were taken just before their scheduled surgeries whereby they would be fasted for at least 8 h for solids and 2 h for plain water as per our institution’s anesthesia requirements.

To find significant perioperative variables to account for the adverse outcomes, a univariate analysis was first performed. Categorical data were analyzed using the chi-squared test and continuous data were analyzed using the Kruskal Wallis test. Significant variables were identified as those having a *p*-value of < 0.2, which were then analysed using Firth multivariate logistic regression to calculate the adjusted odds ratio. Significant factors were identified as those having a p-value of < 0.05.

All statistical analyses were done using IBM SPSS version 25.0 (Armonk, NY, USA) and R version 3.4.4. This manuscript adheres to the applicable STROBE guidelines.

## Results

Patient demographics and surgical characteristics are shown in Table 1. A total of 2106 patients were studied. The population consisted of 53.5% males. Seventy percent of patients were of Chinese ethnicity, 19.1% to Malay, 8.5% to Indians, which closely reflects ethnic distribution in Singapore [17]. Overall, the mean age of the surgical population was 63.7 with the mean age of known diabetics at 64.9, undiagnosed diabetics at 64.7 and non-diabetics at 63.0. The mean BMI of our surgical population was 25.4, while the mean BMI in known diabetics, undiagnosed diabetics and non-diabetics was 26.5, 25.6, and 24.9 respectively.

**Fig. 1:**
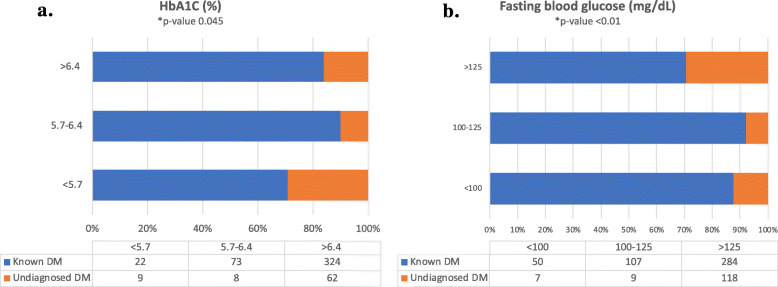
**a** HbA1c levels amongst known and undiagnosed diabetics in the surgical population. **b** Fasting blood glucose levels amongst known and undiagnosed diabetics in the surgical population. DM – diabetes mellitus

**Fig. 2:**
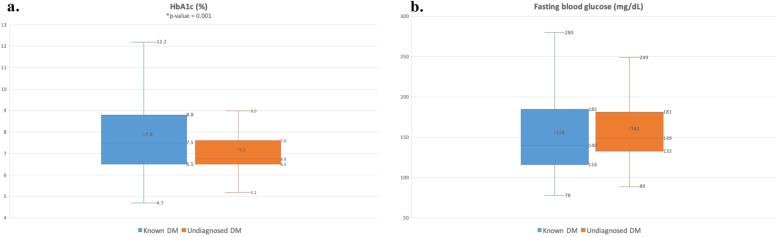
**a** Box and whisker chart of mean and median HbA1c levels amongst known and undiagnosed diabetics. **b** Box and whisker chart of mean and median fasting blood glucose levels amongst known and undiagnosed diabetics. x – mean value; DM – diabetes mellitus

Thirty-six percent (*n* = 757) of the surgical population was diabetic, of which, 79% (*n* = 602) were known diabetics. The prevalence of undiagnosed diabetes in the surgical population was 7.4% (*n* = 155). Among the undiagnosed diabetics, 88% had a fasting blood glucose of > 126 mg/dL and 78.5% had a HbA1c > 6.5 (Fig. 1a and b). The mean and median HbA1c of known diabetics were 7.9 and 7.5%, while the mean and median HbA1c for undiagnosed diabetics were 7.2 and 6.8% respectively (Fig. 2a and b). The mean random blood glucose was 153 mg/dL for the whole population (*n* = 1083), with 36.4% of known diabetics (*n* = 414) and 20.5% of unknown diabetics (*n* = 112) having a random blood glucose > 200 mg/dL.

The patient outcomes of known and undiagnosed diabetics compared with non-diabetics are shown in Table 2. Our results show that undiagnosed diabetics had a three-fold increase in 1-year mortality compared to non-diabetics (adjusted OR 3.46 (1.80–6.49) *p* < 0.001). No statistically significant relationship was found between the known diabetics and one-year mortality. However, the known diabetics were at increased risk for morbidity in terms of increased risks of new onset atrial fibrillation (adjusted OR 2.48 (1.01–6.25) *p* = 0.047), infection (adjusted OR 1.49 (1.07–2.07) *p* = 0.017), readmission within 30 days (adjusted OR 1.62 (1.17–2.25) *p* = 0.004) and of note, 30-day mortality (adjusted OR 3.11 (1.16–8.56) *p* = 0.025).

**Table 2:** Table showing the outcomes of undiagnosed diabetics and known diabetics compared to non-diabetics in both univariate and multivariate regression models

		Univariate analysis		Multivariable analysis	
		Odds ratio (95% Confidence interval)	***p***-value	Adjusted odds ratio (95% Confidence interval)	***p***-value
**30-day readmission**					
Undiagnosed DM^c^	(*n* = 12)	0.98 (0.50–1.78)	0.955	0.80 (0.42–1.54)	0.503
Known DM	(*n* = 83)	1.71 (1.26–2.33)	< 0.001	1.62 (1.17–2.25)	0.004
**30-day infection**					
Undiagnosed DM	(*n* = 25)	2.2 (1.33–3.53)	0.0015	1.26 (0.73–2.11)	0.397
Known DM	(*n* = 89)	1.73 (1.28–2.33)	< 0.001	1.49 (1.07–2.07)	0.017
**30-day mortality**					
Undiagnosed DM	(*n* = 3)	3.50 (0.86–11.2)	0.076	2.15 (0.51–7.38)	0.273
Known DM	(n = 10)	2.44 (1.00–6.03)	0.050	3.11 (1.16–8.56)	0.025
**1-year mortality**					
Undiagnosed DM	(*n* = 18)	4.86 (2.64–8.66)	< 0.001	3.46 (1.80–6.49)	< 0.001
Known DM	(*n* = 23)	1.25 (0.74–2.09)	0.400	1.17 (0.66–2.02)	0.587
**New onset atrial fibrillation**					
Undiagnosed DM	(n = 4)	4.54 (1.31–13.6)	0.020	2.52 (0.69–8.01)	0.151
Known DM	(n = 12)	2.91 (1.25–7.00)	0.013	2.48 (1.01–6.25)	0.047

^c^*DM* Diabetes mellitus

## Discussion

Undiagnosed diabetes is a significant and worrying epidemic with a prevalence of 7.4% in our Asian surgical population. The mean HbA1c and proportion of patients with random blood glucose > 200 mg/dL are lower in the undiagnosed diabetics than the known diabetics, suggesting that undiagnosed diabetics have a biochemically less severe form of diabetes at the point of testing. Despite this, our results indicated that undiagnosed diabetics had a three-fold increase in 1-year mortality compared to non-diabetics – a mortality disadvantage that was not seen in known diabetics.

Even in the non-surgical population, patients with prediabetes have been associated with increased risk of morbidity and mortality as shown in a meta-analysis from 53 non-surgical studies [18]. A recent Korean cohort study of almost 500,000 non-surgical patients suggests that the risk could be amplified in the Asian population due to different adiposity distributions from the Westerners [19, 20]. Therefore, it can be postulated that if patients with prediabetes are already at risk, underdiagnosed diabetics with greater biochemical derangement undergoing the stress of surgery would be at an even higher risk.

The study highlights a second worrying trend of this silent killer; that known diabetics in our population are not well controlled in the perioperative setting. The mean HbA1c of the known diabetic group was 7.9%, with more than 36.4% of the group having a random blood glucose > 200 mg/dL. This has significant postoperative consequences. Our study found that known diabetics had a 1.5-fold and 2.5-fold increase in risks of adverse outcomes including postoperative infection and new onset atrial fibrillation respectively, which likely explains the increased 30-day readmission and mortality rates. However, this did not lead to more deaths at 1 year, which could be attributed to effective disease surveillance, follow-up and treatment.

Our finding of infective complications with poor diabetic control concurs with a recent meta-analysis which revealed that diabetics undergoing elective total knee replacement surgeries had increased risks of deep infection amongst other complications [21, 22]. This is in line with biological research whereby immune dysfunction occurs in a hyperglycemic environment resulting in impaired neutrophil function, altered chemotaxis and phagocytic activity, as well as impaired wound healing due to delayed collagen synthesis [23].

The finding of increased incidence of atrial fibrillation in diabetic patients with poor glucose control is in accordance with other large population-based cohort studies [24–26]. Development of atrial fibrillation could be a consequence of systemic inflammation or autonomic stimulation perioperatively in a susceptible myocardium with other predisposing factors like electrolyte abnormalities. Diabetes is postulated to cause structural, electromechanical and autonomic remodeling of the heart which increases the propensity for atrial fibrillation [27, 28]. Massera et al., studying 80,000 subjects, reported a higher 30-day mortality in patients with new onset atrial fibrillation compared to those without [29].

The increased risks of infection and atrial fibrillation were not seen in the undiagnosed diabetics in our surgical population. This is likely because known diabetics in our study population generally have a more severe form of the disease than the undiagnosed diabetics at the point of biochemical testing. This is evident by the higher mean HbA1c (7.9% in known diabetics vs 7.2% in undiagnosed diabetics)*.* Closer follow-up and higher index of suspicion for complications among known diabetics could account for the higher pick-up rates of infection, arrhythmia and readmission in our study population. On the other hand, because undiagnosed diabetics remain untreated and are not actively followed up, complications could progress, resulting in a significantly higher one-year mortality rate. This finding is compelling and highlights the importance of screening and treatment for patients aged 45 years and above presenting for elective surgery, with benefits that extend beyond the perioperative period to their subsequent general state of health and wellbeing.

A successful preoperative diabetic screening program cited by the American Diabetic Association is a diabetic clinic nested within a preoperative clinic. In the intervention group, patients with HbA1c of > 8% are identified and treated. This saw a preoperative reduction of a mean HbA1c from 9.5 to < 7%, with no increase in hypoglycemia or complications [30]. We therefore advocate implementation of similar multidisciplinary clinics. Early referrals for urgent optimization of newly diagnosed and poorly controlled diabetes will minimize delay in surgery. While the UK NHS’ ‘Super Six’ model of care is lauded for including antenatal diabetes and diabetic nephropathy into primary care support, we believe that a seventh specialist diabetes care area could extend to the perioperative setting [31]. It is clear that it is the role of the perioperative physician to take ownership and collaborate timely care planning amongst patients, primary and specialist care teams. In hospital, the same stringent diabetic protocol (i.e. close monitoring and optimization of blood sugar levels, usage of an insulin sliding scale, strict diet control, screening for complications) should be implemented on newly diabetic patients.

We acknowledge limitations with a retrospective cohort study that may have selected for sicker patients with a higher index of suspicion of hyperglycemia to be screened for diabetes. The robustness of follow-up and treatment of diabetes post-surgery could not be studied as well. Some of our surgical population had no fasting blood glucose or HbA1c done which likely underestimates the prevalence of undiagnosed diabetes. However, this would serve to emphasize the magnitude of the issues highlighted in our study. Moreover, as HbA1c and fasting blood glucose are not routine pre-operative investigations by most clinical guidelines, our results are likely to be a true reflection of world-wide perioperative practice and outcomes.

Further studies would be needed to establish risk factors specific to the Asian population that are associated with undiagnosed diabetes. This would have the advantage of streamlining the screening process. A subsequent step would then be the need to conduct formal cost effectiveness analysis or ascertain the magnitude of adverse outcome reduction and ensure that a screening program is financially viable when balanced against health expenditures due to projected complications.

## Conclusion

Undiagnosed diabetes is a prevalent problem in our Asian surgical population. It is associated with a significantly increased risk of one-year mortality compared to non-diabetics. This mortality disadvantage is not seen with the known diabetics, despite being biochemically more severe compared to the unknown diabetics at the point of testing.

Overall, this may justify opportune diabetes screening for patients, especially non-compliant patients, aged 45 years old and above, presenting for elective surgery without additional healthcare visits, fasting, or venepunctures. This may allow medical intervention and lifestyle modification at an earlier stage of disease. With effective surveillance, follow-up and treatment, this may reduce the social and economic burden of untreated diabetes in the long term.

## Data Availability

All data generated or analysed during this study are included in this published article.

## Abbreviations

ASA: American Society of Anaesthesiologists physical status class
BMI: Body mass index
DM: Diabetes mellitus
HbA1c: Glycated haemoglobin
ICD-9: International Classification of Diseases, Ninth Revision
